# Impact of PCSK9 inhibitor on T lymphocyte subsets and cytokines in patients with acute ischemic stroke: an exploratory analysis of a randomized clinical trial

**DOI:** 10.3389/fneur.2025.1688553

**Published:** 2025-11-24

**Authors:** Jiahui Liu, Chao Han, Xiaofei Ji, Hua Cao, Shuna Chen, Chengcheng Tan, Xinqing Hao, Yuki Joyama, Wei Zou, Ying Li, Jing Liu

**Affiliations:** 1 Stem Cell Clinical Research Center, National Joint Engineering Laboratory, Regenerative Medicine, The First Affiliated Hospital of Dalian Medical University, Dalian, China; 2 Dalian Innovation Institute of Stem Cell and Precision Medicine, Dalian, China; 3 Department of Neurology, The First Affiliated Hospital of Dalian Medical University, Dalian, China; 4 Department of Neurology, The People's Hospital of Naqu, Naqu, China; 5 Department of Gastroenterology and Hepatology, Tokyo Medical University, Tokyo, Japan

**Keywords:** acute ischemic stroke, early neurological deterioration, PCSK9 inhibitors, evolocumab, T lymphocyte subsets, T cell compartments, cytokines

## Abstract

**Background:**

PCSK9 inhibitors are lipid-lowering agents with pleiotropic properties including immune-inflammatory modulation. However, the effects of PCSK9 inhibitors on the peripheral immune profile of patients with acute ischemic stroke (AIS) remain unknown. In this analysis, we aim to further investigate the impact of PCSK9 inhibitor evolocumab on clinical outcomes, immune responses, and cytokines in AIS patients.

**Methods:**

In this study, a total of 100 patients with AIS were included for the current analysis (*n* = 50 for combination therapy of evolocumab and atorvastatin, PI group, *n* = 50 for atorvastatin monotherapy, AT group). Blood samples were collected at baseline and 7 days after evolocumab administration. T lymphocyte subsets, T helper (Th) cell subsets, and T cell compartments were identified by flow cytometry. The concentrations of interleukin-6 (IL-6), interleukin-8 (IL-8), and tumor necrosis factor-a (TNF-a) were also measured.

**Results:**

Compared with the AT group, patients in the PI group had a significantly lower incidence of early neurological deterioration (END) (*p* = 0.032). Also, the PI group had a significantly higher proportion of favorable functional outcomes at 90 days than the AT group (*p* = 0.001). Moreover, the plasma IL-6 concentration was significantly lower in the PI group than in the AT group at 7 days after treatment (*p* = 0.023). However, the T lymphocyte subsets, Th cell subsets, and T cell compartments were not statistically different between the two groups at baseline or 7 days after treatment (*p* > 0.05).

**Conclusions:**

This study demonstrated that adjunctive evolocumab therapy significantly improved clinical outcomes and inhibited the elevation of the plasma IL-6 compared to atorvastatin monotherapy in AIS patients, whereas peripheral blood T lymphocyte subsets did not change in this trial.

**Trial registration:**

http://www.chictr.org.cn; Identifier: ChicTR2200059445. Date of registration: 29 April 2022.

## 1 Introduction

Ischemic stroke (IS) is one of the leading causes of disability and death, exerting a heavy burden on individuals, families, and society (1). Current acute-phase therapy prioritizes revascularization strategies, including intravenous thrombolysis and mechanical thrombectomy. However, only a small portion of patients can receive these treatments due to the narrow time window (2). Despite hundreds of neuroprotective drugs providing promising preclinical evidence, few have been successfully translated into clinical application (3, 4). Thus, novel treatment strategies are keenly required.

The immune responses play an essential role in the pathogenesis and progression of IS (5). Therefore, the study of modifiable factors influencing the immune response in IS is vital to improve patients' prognosis. During the pathophysiological cascade of ischemic stroke, hypoxia at the injury site triggers autoimmune reactivity against neural antigens, followed by the activation of resident neuroglial populations, particularly astrocytes and microglia, to secrete pro-inflammatory cytokines including interleukin-1β (IL-1β), interleukin-6 (IL-6), and tumor necrosis factor-a (TNF-a). These cytokines then mobilize peripheral immune cells (neutrophils, monocyte-derived macrophages, T and B cells) to transmigrate across the compromised blood-brain barrier (BBB) and infiltrate the brain parenchyma, thereby exacerbating neuroinflammation (6). Among these immune components, T cells have been extensively characterized for their dual roles in mediating both innate and adaptive immune responses (7). Studies have demonstrated that T cells infiltrate within the first 24 h after a stroke and might persist for years to participate in immune responses (8, 9). Current classification systems stratify T cells into two principal subsets based on surface markers: CD4^+^ helper T (Th) cells and CD8^+^ cytotoxic T (CTL) cells, which exhibit differential pathophysiological roles in acute ischemic stroke (AIS) (10). Furthermore, CD4^+^ T cells can be further activated and differentiated into distinct subsets, including Th1, Th2, Th17, and regulatory T cells (Tregs), each of which promotes different effector functions through various antigenic stimuli and cytokine signals (11). Thus, the dual properties of T cells make them play a key role in injury and repair after stroke (12).

Proprotein convertase subtilisin/kexin type 9 (PCSK9), a plasma protein secreted by hepatocytes, has been principally involved in cholesterol metabolism. Recent advances have unveiled the pleiotropic properties of PCSK9 with emphasis on the newly recognized immunomodulatory functions (13, 14). This emerging property appears to be another reason why PCSK9 inhibitors can reduce cardiovascular risk in addition to their well-documented effect on lowering circulating low-density lipoprotein cholesterol (LDL-C) levels. The formation and progression of atherosclerotic plaques are always accompanied by inflammatory response (15). Recent studies indicated the potential role of PCSK9 in the differentiation of T cells. Seminal work by Liu et al. showed that the presence of T cells and dendritic cells (DCs) in atherosclerotic plaques might play an important role in the development of atherosclerosis (16). Subsequent mechanistic investigations further suggested the immunological role of PCSK9 in oxidized LDL (OxLDL)-induced DCs maturation and subsequent T cell activation (17). Specifically, PCSK9 overexpression promoted the polarization of naive CD4^+^ T cells to pro-inflammatory Th1 and Th17 subpopulations, whereas PCSK9 knockdown induced the polarization of naive CD4^+^ T cells to Treg cells, which may contribute to reduced inflammation and a favorable prognosis of cardiovascular disease, independent of LDL-C lowering (17). Consistently, another study reported a significant reduction in Th17 cells in mice lacking PCSK9, and the results indicated that PCSK9 was associated with T cell programming contributing to the development of atherosclerosis (18). Collectively, these findings suggested that PCSK9 exerted immunomodulatory properties in atherosclerotic plaques and human blood through distinct molecular mechanisms, providing an immunological role for atherosclerosis attenuation beyond LDL-lowering.

Although preclinical studies have elucidated PCSK9′ regulatory effects on T lymphocyte proliferation and differentiation, clinical evidence from randomized trials exploring the impact of PCSK9 inhibitors on the T lymphocyte subsets remains scarce. Thus, we performed a sub-analysis of a randomized trial to investigate the impact of PCSK9 inhibitor evolocumab on circulating T lymphocyte subsets and cytokines in AIS patients. T lymphocyte subsets were identified by flow cytometry, and plasma cytokines concentrations were measured by the IMMULITE 2000 immunoassay system.

## 2 Materials and methods

### 2.1 Study design and population

This study is an exploratory sub-analysis of a randomized trial designed to test the impact of evolocumab on peripheral immunoinflammatory responses in AIS. The detailed study design and major results of the parent trial have been reported previously (19). In brief, the previous study was a multicenter, randomized trial with blinded outcome assessments to evaluate the efficacy and safety of evolocumab in preventing early neurological deterioration (END) for AIS patients.

Eligible patients were aged 18–80 years, diagnosed with acute non-cardiogenic ischemic stroke within 24 h after stroke onset, and had been functioning independently before stroke with modified Rankin Scale (mRS) scores ≤ 1. Exclusion criteria included hemorrhagic or mixed stroke, receiving intravenous thrombolysis or endovascular therapy, cardiogenic embolism, any contraindication for statin treatment, and PCSK9 inhibitors use within 3 months before the stroke onset. The parent study included a total of 272 patients. Enrolled patients were randomly assigned to two groups: the PI group, which received evolocumab 140 mg subcutaneously combined with atorvastatin 40 mg orally within 24 h of symptom onset, followed by maintenance therapy with biweekly evolocumab 140 mg and atorvastatin 40 mg once a night from day 2 to day 90. The AT group received atorvastatin 40 mg orally once a night throughout the 90-day trial period. All participants received protocolized antiplatelet therapy per our published methodology (19).

The primary endpoint of the parent study was the incidence of END at 7 days and the results have been published. In this trial, END was defined as an increase of ≥ 2-point in the National Institutes of Health Stroke Scale (NIHSS) score or ≥ 1-point in the NIHSS motor score within 7 days of admission compared with the baseline. The present analysis is a sub-study of the parent trial in which we randomly selected peripheral blood samples from 100 patients from the total study population for T lymphocyte subsets and cytokines analysis. The present analysis is a sub-study of the parent trial. Peripheral blood samples were randomly selected from 100 patients within the overall study population for T lymphocyte subsets and cytokines analysis. Randomization was performed using computer-generated random number sequences produced by SPSS software version 26.0. Blood samples were collected at two time points: baseline and 7 days after the study intervention. The baseline blood samples were collected at the time of randomization (within 24 h of AIS symptom onset). The post-treatment samples were collected on day 7 following the study intervention. As an exploratory endpoint, we aimed to further explore the impact of PCSK9 inhibitor evolocumab on peripheral immunoinflammatory profile in patients with AIS.

The study was approved by the Ethics Committee of the First Affiliated Hospital of Dalian Medical University and registered in the Chinese Clinical Trial Registry (ChicTR2200059445). All individuals provided written informed consent, and all methods complied with the Declaration of Helsinki.

### 2.2 Clinical data collection

Demographic and clinical characteristics of the enrolled patients were systematically obtained from the medical records. Laboratory tests and clinical scale assessments were performed at baseline and or during follow-up (7 and 90 days after enrollment): (1) Profile of blood lipids, including total cholesterol (TC), triglycerides (TG), low-density lipoprotein cholesterol (LDL-C), and high-density lipoprotein cholesterol (HDL-C); (2) Routine blood test: the counts of leukocytes, neutrophils, lymphocytes, and monocytes, as well as the percentages of neutrophils, lymphocytes, and monocytes, were all examined. The neutrophil-to-lymphocyte ratio (NLR) was based on the ratio of the neutrophil counts to the lymphocyte counts; (3) Serum biochemical parameters, such as fasting blood glucose (FBG) and liver and kidney function tests; (4) Clinical scale assessments: the stroke severity was assessed using NIHSS scores (range 0–42) (20). The disability was measured using mRS scores (range 0–6) (21).

### 2.3 Flow cytometry identification of T lymphocyte subsets

Peripheral venous blood (5 ml) anticoagulated with EDTA was collected at baseline and 7 days post-intervention. To determine the patient's T cell immunophenotyping, the whole blood sample was mixed gently and incubated with fluorochrome-conjugated monoclonal antibodies in darkness at room temperature (25 °C) for 30 min. Then, Erythrocyte lysis (BD Pharm Lyse, USA) was performed following the manufacturer's protocol. After centrifugation for 1,500 rpm, 5 min, the cells were washed twice with 1 mL phosphate buffer solution (PBS) and resuspended in 300 μl PBS. Finally, samples were detected by flow cytometer (SH800S, Sony, Japan), and data were analyzed by using FlowJo software (Treestar, Inc., Ashland, OR, USA). T lymphocyte immunophenotyping was performed using standardized flow cytometry panels adapted from previous publications (22). The antibody cocktail composition (including fluorochrome conjugates, clone numbers, and manufacturers) is detailed in Supplementary Table 1, and the gating strategy is as previously reported by our research group (23). Flow cytometry quantified circulating T-cell subsets using the following panels:

T cell subsets: Helper T lymphocytes (Th): CD45^+^CD3^+^CD4^+^; Cytotoxic T lymphocytes (Ts): CD45^+^CD3^+^CD8^+^;

Th cell subsets: Th1: CXCR3^+^CCR6^−^; Th2: CXCR3^−^CCR6^−^; Th17: CXCR3^−^CCR6^+^; Treg: CD25^+^CD127^low^;

T cell compartments: Naive T (T_N_) cells: CD3^+^CD45RA^+^CCR7^+^; Central memory T (T_CM_) cells: CD3^+^CD45RA^−^CCR7^+^; Effector memory T (T_EM_) cells: CD3^+^CD45RA^−^CCR7^−^; Terminally differentiated effector memory T (T_EMRA_) cells: CD3^+^CD45RA^+^CCR7^−^.

### 2.4 Measurement of plasma cytokines

For plasma collection, whole blood was centrifuged (3,000 rpm, 4 °C, 15 min), and plasma samples were immediately stored at−80 °C. The concentrations of cytokines (IL-6, IL-8, and TNF-α) were measured by the IMMULITE 2000 immunoassay system (Siemens, Deerfield, USA), following the manufacturer's protocols. All analyses were conducted by a certified clinical laboratory technologist.

### 2.5 Statistical analysis

Based on prior studies evaluating analogous immunological outcomes, differences in T lymphocyte subsets between groups were assumed to approximate a medium effect size (Cohen's *d* = 0.5) (24). A two-sided α level of 0.05 with 80% statistical power was performed for statistical tests. To account for potential attrition bias of approximately 10%, the final calculated sample size was a minimum of 100 patients (50 per arm) using the PASS software version 15 (NCSS, LCC, Taiwan 2017). Continuous variables were expressed as mean ± standard deviation (SD) or median with interquartile ranges (IQR). In contrast, categorical variables were expressed as frequencies (percentages). The distribution normality of the continuous variables was evaluated by the Shapiro-Wilk test. The independent samples *t*-test or Mann–Whitney *U* test was used to compare the continuous variables between two groups, and the Chi-square test or Fisher's exact test was used to compare categorical variables between two groups. All analyses were performed with SPSS software version 26.0 (IBM Corp., Armonk, NY, USA) and GraphPad Prism version 9.0 (GraphPad Software Inc., USA). Statistical significance was defined as a 2-sided *p* < 0.05.

## 3 Result

### 3.1 Study population and baseline characteristics

In this substudy, a total of 100 patients completed the study and were included in the analysis, with 50 patients in the PI group and 50 patients in the AT group. Table 1 summarizes the demographic and baseline clinical characteristics of enrolled participants. The analysis showed that there were no significant differences in baseline data between the PI group and AT group, including age, sex, blood pressure, medical history, prior statin use, dual antiplatelet therapy, LDL-C levels, and FBG levels at admission (*p* > 0.05). Furthermore, we compared baseline characteristics and clinical outcomes between the 100 patients included in this analysis and the 172 patients excluded. There were no statistically significant differences between the groups for any baseline characteristics or outcome measures (Supplementary Table 2).

**Table 1:** Demographic and clinical characteristics of the patients at baseline.

**Characteristics**	**PI group (*n* = 50)**	**AT group (*n* = 50)**	***p***
**Age, median (IQR), y**	68 (62, 72)	69 (62, 73)	0.548
**Sex, Female**, ***n*** **(%)**	12 (24.0)	12 (24.0)	1.000
**Medical history**, ***n*** **(%)**			
Hypertension	35 (70.0)	38 (76.0)	0.499
Diabetes	23 (46.0)	16 (32.0)	0.151
Coronary heart disease	7 (14.0)	13 (26.0)	0.134
Prior ischemic stroke	15 (30.0)	10 (20.0)	0.248
Smoking history	27 (54.0)	22 (44.0)	0.317
Current drinker	20 (40.0)	14 (28.0)	0.205
**Regular use of statins before onset**, ***n*** **(%)**	5 (10.0)	8 (16.0)	0.372
**Blood pressure at randomization, (mean** **±SD), mm Hg**			
Systolic	155.70 ± 20.93	158.40 ± 19.60	0.507
Diastolic	90.66 ± 13.42	91.40 ± 13.21	0.782
**NIHSS score at randomization, median (IQR)**	4 (2, 5)	3 (1, 5)	0.649
**mRS score at randomization, median (IQR)**	2 (1, 3)	2 (1, 3)	0.966
**Dual antiplatelet therapy**, ***n*** **(%)**^**a**^	38 (76.0)	31 (62.0)	0.130
Total cholesterol, (mean ± SD), mmol/L	4.74 ± 1.28	4.71 ± 1.18	0.909
LDL-C, (mean ± SD), mmol/L	2.60 ± 0.84	2.70 ± 0.77	0.560
HDL-C, (mean ± SD), mmol/L	1.14 ± 0.27	1.16 ± 0.23	0.625
FBG, median (IQR), mmol/L	6.81 (5.55, 8.59)	5.75 (5.08, 8.38)	0.116

Data are expressed as mean ± SD, median (IQR), or frequency (percentage). PI group, evolocumab plus statin therapy group; AT group, stain monotherapy group; NIHSS, National Institute of Health Stroke Scale; mRS, modified rankin scale; LDL-C, low-density lipoprotein cholesterol; HDL-C, high-density lipoprotein cholesterol; FBG, fasting blood glucose; SD, standard deviation; IQR, interquartile range. ^a^Aspirin combined with clopidogrel.

### 3.2 Clinical outcome

**Figure 1:**
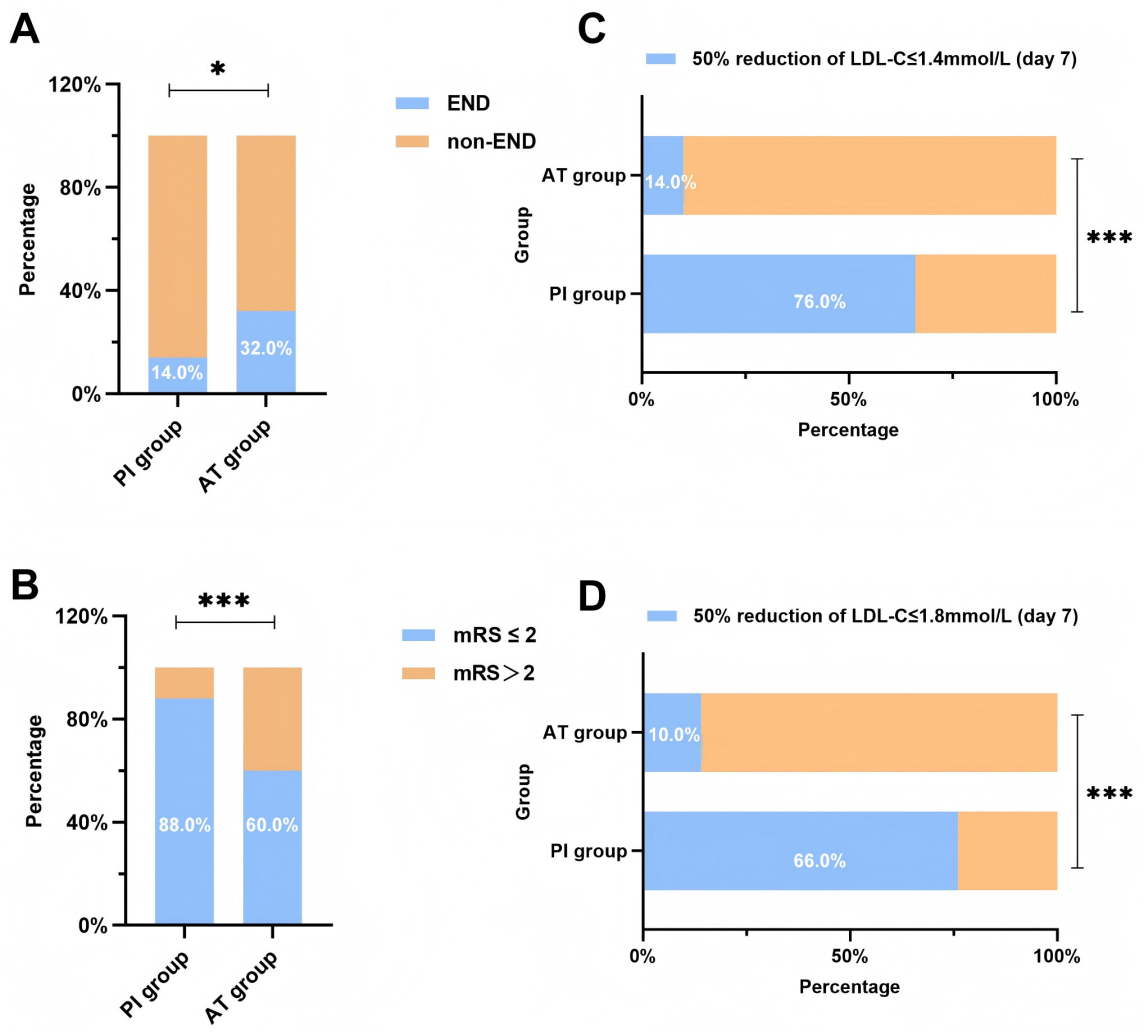
Clinical outcomes comparison between the PI and AT groups. **(A)** Occurrence of END between groups, **(B)** proportion of mRS scores ≤ 2 at day 90 between groups, **(C)** the LDL-C target achievement rate on day 7 (LDL-C ≤ 1.8 mmol/L with a reduction > 50% from baseline), **(D)** the LDL-C target achievement rate on day 7 (LDL-C ≤ 1.4 mmol/L with a reduction > 50% from baseline). Data are presented as mean ± SD or median (IQR). *p < 0.05, **p < 0.01, and ***p < 0.001. END, early neurological deterioration; LDL-C, low-density lipoprotein cholesterol; mRS, modified Rankin scale; PI group, evolocumab plus statin therapy group; AT group, stain monotherapy group.

Figure 1 presents the comparisons of clinical outcomes between the PI and AT groups. Compared with the AT group, patients in the PI group had a significantly lower incidence of END (14.0% vs. 32.0%; RR, 0.79; 95% CI, 0.63 to 0.99; *p* = 0.032; Figure 1A). Also, the PI group had a significantly higher proportion of favorable functional outcomes (mRS score 0–2) at 90-day follow-up than the AT group (88.0% vs. 60.0%; RR, 3.33; 95% CI, 1.46 to 7.60; *p* = 0.001; Figure 1B). In terms of LDL-C target achievement rate at 7 days (defined as LDL-C ≤ 1.8 mmol/L with a reduction > 50% from baseline), there was a significant difference between the PI and AT groups (76.0% vs. 14.0%; RR, 3.58; 95% CI, 2.16 to 5.94; *p* < 0.001; Figure 1C). This therapeutic advantage remained when applying stricter criteria of LDL-C ≤ 1.4 mmol/L with a reduction of >50% from baseline (66.0% vs. 10.0%; RR, 2.65; 95% CI, 1.78 to 3.94; *p* < 0.001; Figure 1D). The statistical results are shown in Supplementary Table 3.

### 3.3 Hematological parameters analysis

**Figure 2:**
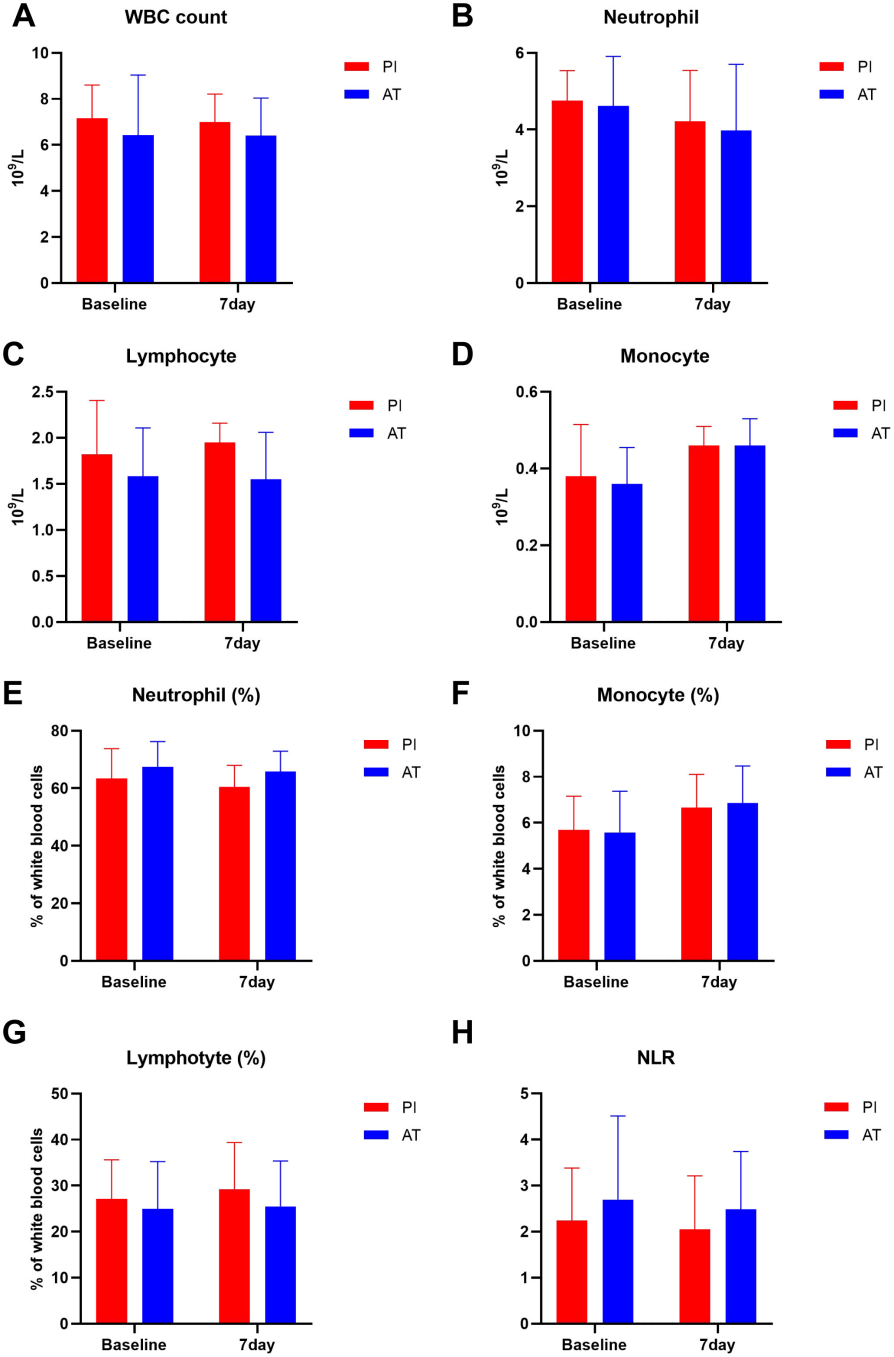
Comparison of leukocyte subtypes in the PI and AT groups at baseline and 7 days following treatment. **(A)** WBC count, **(B)** neutrophil count, **(C)** lymphocyte count, **(D)** monocyte count, **(E)** neutrophil percentage, **(F)** lymphocyte percentage, **(G)** monocyte percentage, and **(H)** neutrophil to lymphocyte ratio. Data are presented as mean ± SD or median (IQR). *p < 0.05, **p < 0.01, and ***p < 0.001. WBC, white blood cell; NLR, neutrophil to lymphocyte ratio.

Figure 2 shows the changes in hematological parameters (including leukocytes, neutrophils, lymphocytes, and monocytes) in two treatment groups. At baseline and 7-day follow-up, the differences in the absolute counts of leukocytes, neutrophils, lymphocytes, monocytes, and NLR were not statistically significant between the PI and AT groups (*p* > 0.05) (Figures 2A–H). Notably, a trend toward increased lymphocyte counts was observed in the PI group at day 7 follow-up [1.95 (1.47, 2.16) × 10^9^/L vs. 1.55 (1.26, 1.97) × 10^9^/L; *p* = 0.051; Figure 2C], though this difference did not reach predefined statistical significance. The statistical results are shown in Supplementary Table 4.

### 3.4 Flow cytometry analysis

**Figure 3:**
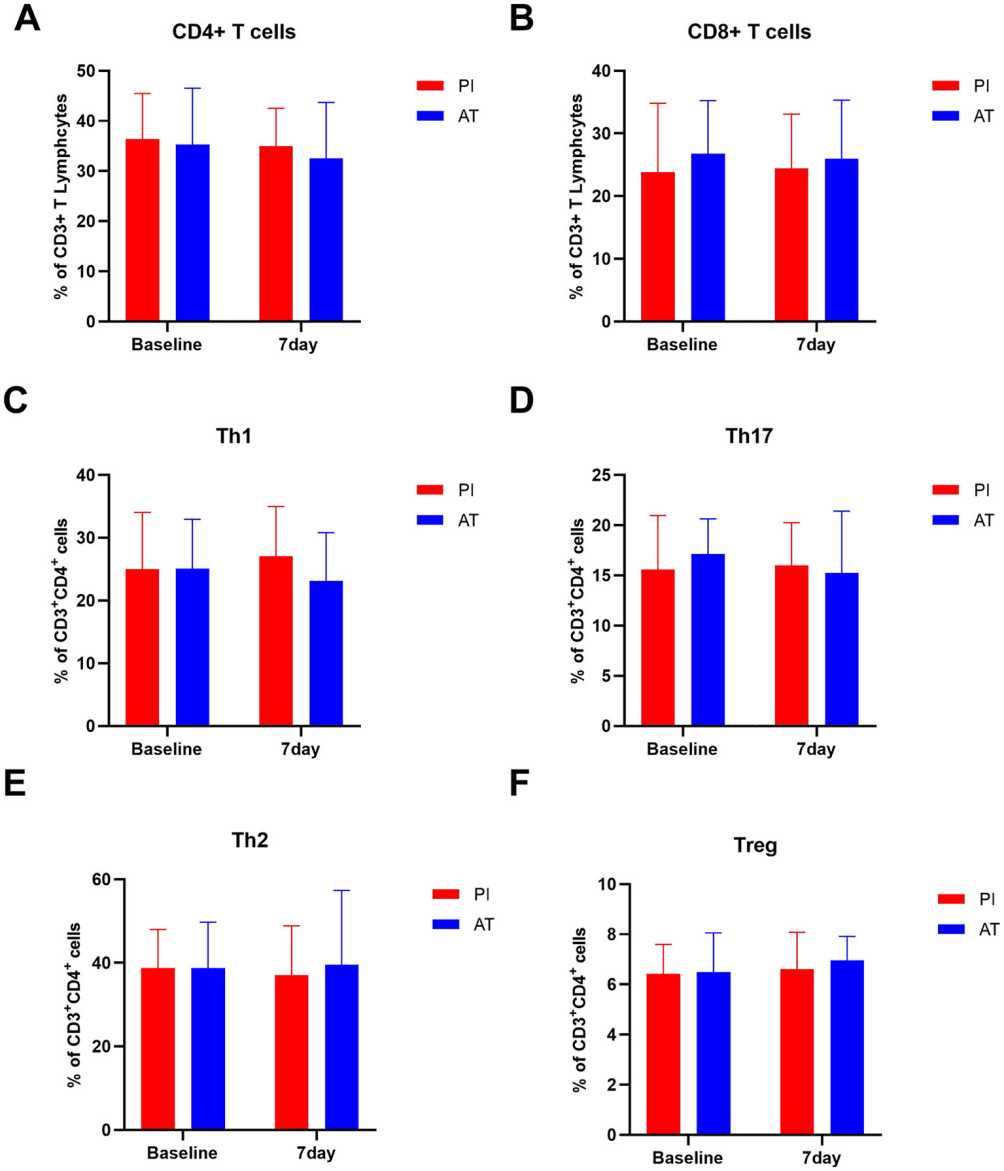
Comparison of T lymphocyte subsets in the PI and AT groups at baseline and 7 days following treatment. **(A)** CD4^+^ T cells, **(B)** CD8^+^ T cells, **(C)** Th1 cells, **(D)** Th2 cells, **(E)** Th17 cells, and **(F)** Treg cells. Data are presented as mean ± SD or median (IQR). Data are expressed as percentages of total T cells (A and B). Data are expressed as percentages of total CD4^+^ T cells (C to F). *p < 0.05, **p < 0.01, and ***p < 0.001. Treg cells, regulatory T cells.

To detect the heterogeneity of peripheral T cells, flow cytometric analysis was conducted. Figure 3 shows the change in T lymphocyte subsets and Th cell subsets over time for the two groups. We found that T lymphocyte subsets (including CD4^+^ T and CD8^+^ T cells within the T cell population) and Th cell subsets (including Th1, Th2, Th17, and Treg cells within the Th cell population) were not statistically different between the two groups at baseline or 7 days after treatment (*p* > 0.05). The statistical results are shown in Supplementary Table 5.

**Figure 4:**
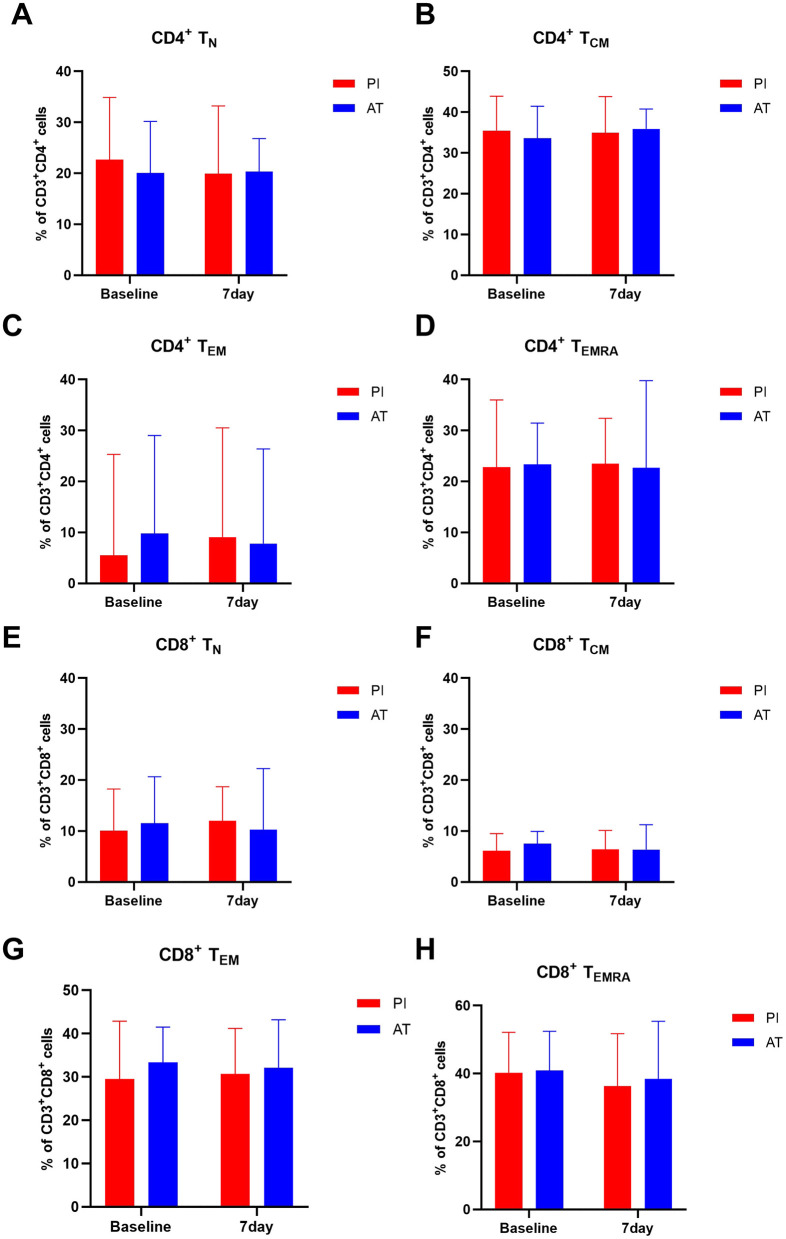
Comparison of T cell compartments in the PI and AT groups at baseline and 7 days following treatment. **(A)** CD4^+^ T_N_, **(B)** CD4^+^ T_CM_, **(C)** CD4^+^ T_EM_, **(D)** CD4^+^ T_EMA_, **(E)** CD8^+^ T_N_, **(F)** CD8^+^ T_CM_, **(G)** CD8^+^ T_EM_, and **(H)** CD8^+^ T_EMRA_. Data are presented as mean ± SD or median (IQR). Data are expressed as percentages of total CD4^+^ T or CD8^+^ T cells. *p < 0.05, **p < 0.01, and ***p < 0.001. T_N_ cells, naive T cells; T_CM_ cells, central memory T cells; T_EM_ cells, effector memory T cells; T_EMRA_ cells, terminally differentiated effector memory T cells.

To further investigate the characteristics of T cell compartments after stroke, naïve T cells (T_N_), central memory T cells (T_CM_), effector memory T cells (T_EM_), and terminally differentiated effector memory T cells (T_EMRA_) of CD4^+^ and CD8^+^ T cells were analyzed. Figure 4 depicts the changes in CD4^+^ and CD8^+^ T cell compartments over time in two groups. There were no significant differences in CD4^+^ or CD8^+^ T cell compartments (T_N_, T_CM_, T_EM_, and T_EMRA_ cells) between groups at baseline or 7 days after treatment (*p* > 0.05). The statistical results are shown in Supplementary Table 5.

### 3.5 Cytokine concentrations of IL-6, IL-8, and TNF-α

To investigate whether evolocumab has an impact on immunoinflammatory markers, we quantified the plasma concentrations of IL-6, IL-8, and TNF-α at baseline and 7 days after treatment. The concentration of IL-6 was significantly lower in the PI group than in the AT group at 7 days after treatment [4.62 (3.04, 12.0) vs. 8.32 (4.24, 19.3) (pg/ml); *p* = 0.023], while the concentrations of IL-8 and TNF-a were not significantly different between the two groups at baseline or 7 days after treatment (*p* > 0.05) (Table 2).

**Table 2:** Comparison of plasma cytokine concentrations in the PI and AT groups.

**Concentration of cytokines**	**Time**	**PI group (*n* = 50)**	**AT group (*n* = 50)**	**Difference (95% CI)**	***p***
IL-6 (pg/ml)	Baseline	4.67 (2.70, 9.30)	4.74 (2.95, 6.29)	−0.18 (−1.68, 0.81)	0.676
	Day 7	4.62 (3.04, 12.0)	8.32 (4.24, 19.3)	2.23 (0.29, 5.25)	**0.023**
IL-8 (pg/ml)	Baseline	63.85 (24.60, 204.00)	48.30 (20.40, 194.00)	−6.10 (−34.20, 14.60)	0.506
	Day 7	67.60 (34.90, 168.00)	50.85 (30.90, 212.00)	−1.75 (−24.90, 20.50)	0.860
TNF-α (pg/ml)	Baseline	16.10 (8.84, 51.10)	17.45 (10.40, 58.10)	1.27 (−3.74, 6.16)	0.533
	Day 7	20.65 (9.78, 45.10)	21.15 (12.70, 72.80)	1.84 (−5.40, 7.46)	0.519

Data are expressed as mean ± SD or median (IQR). *p* < 0.05 were considered statistically significant. IL, interleukin; TNF, tumor necrosis factor. Bold values indicate statistically significant differences.

## 4 Discussion

The present study demonstrated that early adjunctive therapy with PCSK9 inhibitor evolocumab significantly reduced the incidence of END within 7 days and suppressed the elevation of the plasma IL-6 concentrations in patients with AIS, whereas there were no significant changes in peripheral blood T lymphocyte subsets in this trial. To our knowledge, this study is the first to explore the impact of PCSK9 inhibitors on peripheral blood T lymphocyte subsets in AIS patients through a prospective randomized trial.

The pathophysiological mechanisms of END in AIS are complex and remain incompletely elucidated. As a clinically significant complication, END may arise from a variety of pathophysiological processes, including progression of the initial ischemic core, unstable plaque rupture, malignant cerebral edema, hemorrhagic transformation, and recurrent embolic events (25). Our analysis showed that the combination therapy of evolocumab and atorvastatin resulted in a significantly lower incidence of END compared with atorvastatin monotherapy (14.0% vs. 32.0%; *p* = 0.032). Notably, lipid analysis demonstrated a higher rate of LDL-C target achievement rate at 7 days with the combination therapy compared with atorvastatin monotherapy (76.0% vs. 14.0%; *p* < 0.001). Combining the pathophysiological mechanisms of END and the mechanism of action of PCSK9 inhibitor evolocumab, we suggest that adjunctive evolocumab therapy may partially prevent the occurrence of END by enhancing the lipid-lowering effect and stabilizing plaques. Emerging neuroimaging evidence confirmed this mechanistic hypothesis. Two longitudinal high-resolution magnetic resonance imaging (HR-MRI) studies evaluating intracranial plaque characteristics in AIS patients revealed that the combination therapy of evolocumab and statins significantly improved atherosclerotic plaque stability. A prospective single-arm cohort study demonstrated that combination therapy with evolocumab and high-intensity statins attenuated luminal stenosis in patients with intracranial atherosclerotic stenosis (ICAS) during a 6-month follow-up period (26). Furthermore, a randomized comparative trial confirmed that 12-week combination therapy with evolocumab and moderate-intensity statins achieved greater LDL-C reduction and more pronounced stenosis regression compared with statin monotherapy (27). Taken together, early adjunctive therapy with the PCSK9 inhibitor evolocumab has the potential to block key pathways in END pathogenesis by rapidly achieving intensive lipid-lowering targets and improving atherosclerotic plaque stability.

Accumulating evidence underscores the critical role of immune responses in the pathogenesis and progression of AIS (28, 29). In particular, T lymphocytes have received much attention because of their potential role in innate and adaptive immune responses (7). Functional heterogeneity among T cell subpopulations determines their dual roles in post-stroke pathophysiology. A recent study examined the relationship between peripheral blood lymphocyte subsets and clinical prognosis in AIS patients. They found that total T cell percentage, CD3^+^, and CD4^+^ T cell counts were variables independently associated with the prognosis of patients with AIS (30). The reduction of CD4^+^ or CD8^+^ T cells within 24 h after stroke resulted in a decrease in infarct size. In contrast, Tregs had a protective effect on lowering infarct area and improving neurological function (31, 32). Given recent preclinical studies on PCSK9-mediated T cell differentiation, we first assessed the effects of the PCSK9 inhibitor evolocumab on peripheral blood T lymphocyte subsets in patients with AIS in this analysis. We found that PCSK9 inhibitor evolocumab did not induce statistically significant alterations in peripheral blood T lymphocyte subsets in patients with AIS. This result may be interpreted through the following perspectives: First, the mechanism of action of PCSK9 inhibitor evolocumab centers on hepatic LDL receptor recycling. Although previous studies have shown that PCSK9 could modulate macrophage-driven inflammation in atherosclerosis (33) and play an immunological role in oxLDL-induced DCs maturation and T-cell activation in atherosclerotic plaques and human blood (17), these localized effects may be counterbalanced by systemic inflammatory storm characterized by elevated proinflammatory cytokines (IL-6, TNF-α) in AIS. Second, the time window of observation in this study was 7 days after treatment. This single time-point observation may not be sufficient to detect changes in T-cell immunophenotype. Future investigations should perform serial immune monitoring at multiple time points (days 3, 7, 14, and 28 post-treatment) to capture stage-specific immunophenotypic changes. Finally, although the baseline characteristics were well balanced, potential confounders such as subclinical infections or metabolic comorbidities may mask the immunomodulatory effects of PCSK9 inhibitors. In future expanded cohort, refinement of the stratification analysis may enhance detection sensitivity. Despite the negative results, they suggest that PCSK9 inhibitors may have a threshold effect on the modulation of the immune response after stroke, which needs to be further validated in a larger cohort combining transcriptomics with functional immunoassays.

Notably, emerging evidence emphasizes the critical role of post-ischemic neuroinflammatory cascades which synergistically exacerbate tissue damage and contribute to neurological deterioration (34). IL-6 serves as an important driver of the inflammatory responses in patients with AIS, with increased serum levels demonstrating strong correlations with stroke severity and neurological (35–37). Our findings revealed that the concentration of IL-6 in the PI group was markedly lower than that in the AT group at 7 days after evolocumab administration. This observation suggests that PCSK9 inhibitor evolocumab may reduce the incidence of END by partially inhibiting the release of IL-6 after AIS. Emerging evidence suggests the link between PCSK9 and IL-6. Specifically, PCSK9 promotes the expression of Toll-like receptor 4 (TLR4) by activating the nuclear factor kappa-light-chain enhancer of activated B-cell (NF-κB) signaling, which leads to the expression of proinflammatory cytokines, including IL-6 (38). Thus, blocking PCSK9-mediated NF-κB activation may interfere with the upstream process of inflammatory events in AIS, thereby promoting the extensive suppression of the downstream inflammatory cascade. Experimental studies have confirmed this hypothesis: *in vitro* investigations showed that PCSK9 siRNA suppressed oxLDL-induced inflammatory activation of THP-1-derived macrophages by inhibiting the NF-κB pathway (39). *In vivo* studies in apolipoprotein E (apoE) knockout mice revealed that PCSK9 silencing could inhibit the progression of atherosclerosis by reducing vascular inflammation and limiting activation of the TLR4/NF-κB signaling pathway without changing plasma cholesterol levels (33). The anti-inflammatory potential of PCSK9 inhibitors was further confirmed in preclinical stroke models. A recent animal experiment showed that PCSK9 inhibitor evolocumab improved neurobehavioral functions and reduced cerebral infarct volumes, which may be mediated by attenuating neuroinflammation through activation of the GPNMB/CD44 pathway (40). Clinically, a multicenter observational study demonstrated that PCSK9 inhibitor evolocumab significantly decreased the expression of pro-inflammatory proteins (NLRP3, caspase-1, IL-1β, TNF-α, NF-κB) within human atherosclerotic plaques even in patients with LDL-C levels < 2.56 mmol/L (100 mg/dl). This result suggests that evolocumab may have an anti-inflammatory effect in atherosclerotic plaques partially independent of its LDL-C lowering role (41). Taken together, our findings combined with available experimental and clinical evidence position PCSK9 inhibitor as a promising therapeutic strategy for targeting IL-6-mediated neuroinflammation in AIS. Early intervention with evolocumab could provide dual benefits by addressing both dysregulated lipid metabolism and inflammatory activation during the critical window of post-stroke recovery. In the acute phase of stroke, higher IL-6 levels are detrimental to the immune system. However, the differences in IL-6 levels between groups did not result in significant intergroup differences in T lymphocyte subsets in this trial. This suggests that immunomodulation is compartmentalized, and whether evolocumab tends to target myeloid-derived inflammation rather than lymphoid-derived immunity warrants further investigation. In addition, it is worth exploring whether the combination of PCSK9 inhibitors with immunomodulatory drugs such as IL-6 antagonists synergistically ameliorates immune disorders after stroke.

There were some limitations in this study. Firstly, as a *post-hoc* exploratory sub-analysis of the parent trial, the number of subjects in this study was relatively small. The current findings provide preliminary insight into the impact of PCSK9 inhibitors on T lymphocyte subsets in patients with AIS. Additional studies with larger sample sizes are needed to validate these effects. Secondly, we only assessed peripheral immune cells and cytokines over a short period (7 days), potentially missing later immune dynamics. Future studies need to explore long-term effects. Thirdly, this study did not measure other systemic inflammatory markers such as C-reactive protein (CRP), or cardiac markers such as natriuretic peptides (NPs). Future studies incorporating these markers may facilitate a more comprehensive assessment of the immunomodulatory effects of PCSK9 inhibitors. Finally, we only focus on circulating immune cells, and it is unclear whether the changes in the blood can reflect the changes in the tissues. Future investigations should expand cohort sizes and dynamically assess T-cell subsets and cytokine profiles at different time points (e.g., days 14, 28, and 90) after stroke. Such longitudinal analyses will enable a comprehensive characterization of PCSK9 inhibitors' temporal immunomodulatory effects, describing both short-term responses and sustained immunological outcomes. Furthermore, by integrating multi-omics approaches (Single-cell transcriptomics, TCR repertoire sequencing, and ATAC-seq), the impact of PCSK9 inhibitors on the T-cell transcriptome and epigenetic modifications could be further elucidated.

## 5 Conclusion

In summary, this study demonstrates that adjunctive evolocumab therapy significantly improves clinical outcomes and inhibits the elevation of the plasma IL-6 levels compared with atorvastatin monotherapy in AIS patients, with no significant changes in peripheral blood T lymphocyte subsets. Further larger studies on its long-term effects on immune function are necessary.

## Data Availability

The original contributions presented in the study are included in the article/Supplementary material, further inquiries can be directed to the corresponding authors.
