# Supplementary material for: Impact of PCSK9 inhibitor on T lymphocyte subsets and cytokines in patients with acute ischemic stroke: an exploratory analysis of a randomized clinical trial
Source: Front Neurol. 2025 Nov 24;16:1688553. doi: 10.3389/fneur.2025.1688553 (PMC12682626; doi:10.3389/fneur.2025.1688553)
Supplement: Supplementary file 1 [file Table_1.docx]

**Supplementary Material**

**Supplementary Tables**

**Supplementary Table 1** Staining panels used for flow cytometry analysis.

|  | **Fluorochrome** | **Marker** | **Clone** | **manufacturer** |
| --- | --- | --- | --- | --- |
| Panel 1 | PerCP-cy5.5 | CD45 | 2D1 | BD MultiTEST |
|  | FITC | CD3 | SK7 | BD MultiTEST |
|  | APC | CD4 | SK3 | BD MultiTEST |
|  | PE | CD8 | SK1 | BD MultiTEST |
| Panel 2 | Horizon V450 | CD3 | UCHT1 | BD Horizon |
|  | PerCP-Cy5.5 | CD4 | SK3 | BD Pharmingen |
|  | PE-Cy7 | CCR6 | 11A9 | BD Pharmingen |
|  | PE | CXCR3 | 1C6 | BD Pharmingen |
| Panel 3 | Horizon V450 | CD3 | UCHT1 | BD Horizon |
|  | PerCP-Cy5.5 | CD4 | SK3 | BD Pharmingen |
|  | PE | CD25 | M-A251 | BD Pharmingen |
|  | Alexa 647 | CD127 | HIL-7R-M21 | BD Pharmingen |
| Panel 4 | Horizon V450 | CD3 | UCHT1 | BD Horizon |
|  | PerCP-Cy5.5 | CD4 | SK3 | BD Pharmingen |
|  | APC | CD8 | SK1 | BD Pharmingen |
|  | PE-Cy7 | CD45RA | HI100 | BD Pharmingen |
|  | PE | CCR7 | 150503 | BD Pharmingen |

**Supplementary Table 2** Comparison of clinical characteristics and outcomes in the selected and non-selected groups.

| **Characteristics** | **Selected group**  **(n=100)** | **Non-selected group**  **(n=172)** | ***p*** |
| --- | --- | --- | --- |
| **Age, median (IQR), y** | 68 (62, 72) | 67 (60, 71) | 0.112 |
| **Sex, Female, n (%)** | 24 (24.0) | 52 (30.2) | 0.269 |
| **Medical history, n (%)** |  |  |  |
| Hypertension | 73 (73.0) | 113 (65.7) | 0.212 |
| Diabetes | 39 (39.0) | 66 (38.4) | 0.918 |
| Coronary heart disease | 20 (20.0) | 26 (15.1) | 0.300 |
| Prior ischemic stroke | 25 (25.0) | 35 (20.3) | 0.372 |
| Smoking history | 49 (49.0) | 76 (44.2) | 0.442 |
| Current drinker | 34 (34.0) | 50 (29.1) | 0.396 |
| **Regular use of statins before onset,**  **n (%)** | 13 (13.0) | 12 (7.0) | 0.097 |
| **Blood pressure at randomization, (mean ± SD), mm Hg** | | | |
| Systolic | 157.05 ± 20.22 | 154.90 ± 21.34 | 0.414 |
| Diastolic | 91.03 ± 13.25 | 92.81 ± 14.93 | 0.325 |
| **NIHSS score at randomization,**  **median (IQR)** | 3 (2, 5) | 2 (1, 4) | 0.076 |
| **mRS score at randomization,**  **median (IQR)** | 2 (1, 4) | 2 (1, 3) | 0.392 |
| **Dual antiplatelet therapy, n (%)** | 69 (69.0) | 137 (79.7) | 0.057 |
| Total cholesterol, median (IQR), mmol/L | 4.73 (4.01, 5.54) | 5.02 (4.32, 5.59) | 0.107 |
| LDL-C, (mean ± SD), mmol/L | 2.65 ± 0.80 | 2.81 ± 0.74 | 0.099 |
| HDL-C, median (IQR), mmol/L | 1.17 (0.93, 1.30) | 1.09 (0.96, 1.29) | 0.383 |
| FBG, median (IQR), mmol/L | 6.20 (5.16, 8.60) | 5.83 (5.07, 7.68) | 0.199 |
| **Outcomes, n (%)** |  |  |  |
| END | 23 (23.0) | 29 (16.9) | 0.214 |
| LDL-C (1850%) | 45 (45.0) | 76 (44.2) | 0.896 |
| LDL-C (1450%) | 38 (38.0) | 64 (37.2) | 0.897 |
| mRS ≤ 2 (90 days) | 74 (74.0) | 128 (74.4) | 0.939 |

Data are expressed as mean ± SD, median (IQR), or frequency (percentage). Abbreviations: PI group, evolocumab plus statin therapy group; AT group, statin monotherapy group; NIHSS, National Institute of Health Stroke Scale; mRS, Modified Rankin Scale; LDL-C, low-density lipoprotein cholesterol; HDL-C, high-density lipoprotein cholesterol; FBG, fasting blood glucose; SD, Standard Deviation; IQR, interquartile range; END, early neurological deterioration; 1850%, LDL-C ≤ 1.8 mmol/L with a decrease exceeding 50% of baseline; 1450%, LDL-C ≤ 1.4 mmol/L with a decrease exceeding 50% of baseline.

^a^Aspirin combined with clopidogrel.

**Supplementary Table 3** Clinical outcomes comparison in the PI and AT groups.

| **Outcomes** | **PI group**  **(n = 50)** | **AT group**  **(n = 50)** | **Risk difference (95% CI)** | **Risk ratio**  **(95% CI)** | ***p*** |
| --- | --- | --- | --- | --- | --- |
| END | 7 (14.0) | 16 (32.0) | -0.18  (-0.34 to -0.02) | 0.79  (0.63 to 0.99) | **0.032** |
| LDL-C (1850%) | 38 (76.0) | 7 (14.0) | 0.62  (0.47 to 0.77) | 3.58  (2.16 to 5.94) | **< 0.001** |
| LDL-C (1450%) | 33 (66.0) | 5 (10.0) | 0.56  (0.40 to 0.72) | 2.65  (1.78 to 3.94) | **< 0.001** |
| mRS ≤ 2  (90 days) | 44 (88.0) | 30 (60.0) | 0.28  (0.12 to 0.44) | 3.33  (1.46 to 7.60) | **0.001** |

Abbreviations: END, early neurological deterioration; LDL-C, low-density lipoprotein cholesterol; mRS, modified Rankin scale; PI group, evolocumab plus statin therapy group; AT group, stain monotherapy group; CI, confidence interval; 1850%, LDL-C ≤ 1.8 mmol/L with a decrease exceeding 50% of baseline; 1450%, LDL-C ≤ 1.4 mmol/L with a decrease exceeding 50% of baseline.

**Supplementary Table 4** Comparison of hematological parameters in the PI and AT groups.

|  | **Time** | **PI group (n = 50)** | **AT group (n = 50)** | **Difference**  **(95% CI)** | | ***p*** |
| --- | --- | --- | --- | --- | --- | --- |
| WBC count  (×10^9^/L) | Baseline | 7.15  (6.03,8.59) | 6.43  (5.50, 9.04) | -0.42  (-1.12, 0.30) | 0.263 | |
|  | Day 7 | 7.00 ± 1.70 | 6.83 ± 1.62 | -0.17  (-0.92, 0.58) | 0.652 | |
| Neutrophil  (×10^9^/L) | Baseline | 4.75  (3.52, 5.52) | 4.61  (3.50, 5.83) | -0.08  (-0.67, 0.72) | 0.833 | |
|  | Day 7 | 4.21  (3.25, 5.45) | 3.97  (3.30, 5.43) | 0.00  (-0.72, 0.65) | 0.992 | |
| Lymphocyte  (×10^9^/L) | Baseline | 1.82  (1.53, 2.40) | 1.59  (1.26, 2.09) | -0.21  (-0.46, 0.02) | 0.071 | |
|  | Day 7 | 1.95  (1.47, 2.16) | 1.55  (1.26, 1.97) | -0.28  (-0.54, 0.00) | 0.051 | |
| Monocyte  (×10^9^/L) | Baseline | 0.38  (0.31, 0.51) | 0.36  (0.31, 0.45) | -0.02  (-0.08, 0.02) | 0.329 | |
|  | Day 7 | 0.46  (0.38, 0.51) | 0.46  (0.39, 0.53) | 0.01  (-0.04, 0.06) | 0.669 | |
| Neutrophil (%) | Baseline | 65.27 ± 9.62 | 67.41 ± 12.08 | 2.15  (-2.19, 6.48) | 0.328 | |
|  | Day 7 | 61.24 ± 10.71 | 64.95 ± 11.05 | 3.71  (-1.16, 8.58) | 0.134 | |
| Lymphocyte (%) | Baseline | 27.10 ± 8.50 | 24.94 ± 10.29 | -2.16  (-5.90, 1.59) | 0.256 | |
|  | Day 7 | 29.20 ± 10.15 | 25.47 ± 9.89 | -3.73  (-8.23, 0.78) | 0.104 | |
| Monocyte (%) | Baseline | 5.69 ± 1.46 | 5.58 ± 1.80 | -0.12  (-0.77, 0.53) | 0.720 | |
|  | Day 7 | 6.66 ± 1.44 | 6.85 ± 1.62 | 0.19  (-0.49, 0.87) | 0.579 | |
| NLR | Baseline | 2.25  (1.74, 3.37) | 2.69  (1.75, 4.40) | 0.36  (-0.19, 0.95) | 0.206 | |
|  | Day 7 | 2.05  (1.54, 2.94) | 2.48  (1.94, 3.68) | 0.50  (0.25, 0.78) | 0.148 | |

Data are presented as mean ± SD or median (IQR). *p* < 0.05 were considered statistically significant. Abbreviations: WBC, white blood cell; NLR, neutrophil-to-lymphocyte ratio.

**Supplementary Table 5** Comparison of peripheral blood T lymphocyte phenotype profiles in the PI and AT groups.

|  | **Time** | **PI group (n = 50)** | **AT group (n = 50)** | **Difference**  **(95% CI)** | ***p*** |
| --- | --- | --- | --- | --- | --- |
| **T cell subsets (%)** | | | | | |
| Th cells | Baseline | 37.13 ± 13.43 | 34.78 ± 14.61 | -2.36  (-7.93, 3.21) | 0.403 |
|  | Day 7 | 34.95  (32.00, 42.48) | 32.57  (23.98, 43.22) | -3.09  (-7.87, 1.30) | 0.163 |
| Tc cells | Baseline | 25.64 ± 10.29 | 27.63 ± 9.48 | 1.99  (-1.93, 5.92) | 0.316 |
|  | Day 7 | 25.07 ± 10.12 | 26.69 ± 9.13 | 1.62  (-2.21, 5.44) | 0.403 |
| **Th cell subsets (%)** | | | | | |
| Th1 cells | Baseline | 26.56 ± 11.57 | 25.73 ± 9.05 | -0.83  (-4.96, 3.29) | 0.689 |
|  | Day 7 | 27.72 ± 9.54 | 24.46 ± 8.64 | -3.27  (-6.88, 0.34) | 0.076 |
| Th2 cells | Baseline | 39.40 ± 15.36 | 39.08 ± 11.62 | -0.32  (-6.68, 6.03) | 0.919 |
|  | Day 7 | 37.10  (29.24, 48.76) | 39.59  (32.77, 57.31) | 3.16  (-3.12, 9.53) | 0.363 |
| Th17 cells | Baseline | 15.60  (11.91, 20.95) | 17.12  (12.07, 20.50) | 0.39  (-2.24, 3.00) | 0.705 |
|  | Day 7 | 15.99  (11.28, 20.21) | 15.26  (12.37, 21.32) | 0.57  (-1.94, 3.12) | 0.632 |
| Treg cells | Baseline | 6.43  (5.10, 7.58) | 6.50  (5.44, 8.01) | 0.30  (-0.52, 1.14) | 0.465 |
|  | Day 7 | 6.81 ± 2.13 | 6.75 ± 2.04 | -0.06  (-0.89, 0.77) | 0.868 |
| **CD4^+^ T cell compartments (%)** | | | | | |
| T_N_ cells | Baseline | 22.67  (13.98, 34.80) | 20.05  (12.67, 30.16) | -3.33  (-9.03, 1.94) | 0.220 |
|  | Day 7 | 19.90  (12.54, 32.70) | 20.30  (13.03, 26.66) | -0.71  (-6.39, 4.33) | 0.720 |
| T_CM_ cells | Baseline | 35.46  (29.03, 43.85) | 33.61  (26.96, 41.24) | -1.93  (-6.08, 2.02) | 0.383 |
|  | Day 7 | 34.53 ± 11.92 | 35.21 ± 10.04 | 0.68  (-3.70, 5.05) | 0.676 |
| T_EM_ cells | Baseline | 5.51  (1.32, 24.37) | 9.80  (2.04, 28.64) | 0.79  (-1.25, 5.54) | 0.361 |
|  | Day 7 | 9.08  (2.18, 30.25) | 7.81  (1.77, 26.34) | -0.68  (-5.04, 2.17) | 0.448 |
| T_EMRA_ cells | Baseline | 22.78  (5.25, 35.96) | 23.35  (4.11, 30.41) | -0.62  (-7.56, 5.68) | 0.801 |
|  | Day 7 | 23.49  (4.16, 32.35) | 22.68  (6.52, 39.72) | 2.20  (-2.79, 10.14) | 0.345 |
| **CD8^+^ T cell compartments (%)** | | | | | |
| T_N_ cells | Baseline | 10.09  (6.34, 18.25) | 11.54  (7.03, 20.60) | 1.86  (-1.35, 5.23) | 0.279 |
|  | Day 7 | 12.03  (5.87, 18.31) | 10.30  (6.50, 21.90) | 0.41  (-3.39, 4.03) | 0.842 |
| T_CM_ cells | Baseline | 6.13  (4.16, 9.21) | 7.52  (4.26, 9.65) | 0.61  (-1.06, 2.35) | 0.530 |
|  | Day 7 | 6.38  (4.09, 10.09) | 6.36  (3.62, 10.79) | 0.14  (-1.81, 2.12) | 0.874 |
| T_EM_ cells | Baseline | 29.53  (21.17, 41.76) | 33.37  (28.80, 41.41) | 3.57  (-1.76, 8.64) | 0.187 |
|  | Day 7 | 32.07 ± 14.49 | 33.25 ± 13.77 | 1.18  (-4.43, 6.79) | 0.677 |
| T_EMRA_ cells | Baseline | 41.71 ± 15.54 | 40.70 ± 14.99 | -1.02  (-7.08, 5.04) | 0.740 |
|  | Day 7 | 40.80 ± 16.39 | 41.78 ± 16.36 | 0.98  (-5.52, 7.48) | 0.766 |

Data are expressed as mean ± SD or median (IQR). *p* < 0.05 were considered statistically significant. Abbreviations: Th cells, helper T lymphocytes; Tc cells, cytotoxic T lymphocytes; Treg cells, regulatory T cells; T_EMRA_ cells, terminally differentiated effector memory T cells; T_EM_ cells, effector memory T cells; T_CM_ cells, central memory T cells; T_N_ cells, naïve T cells.
